# Type 2 Diabetes, Visceral Adiposity, and Carotid Intima–Media Thickness in Hospitalized Adults: A Retrospective Cross-Sectional Analysis

**DOI:** 10.3390/medicina62091690

**Published:** 2026-09-03

**Authors:** Alina Andreea Tischer, Loredana Gabriela Stana, Petra Alexandra Lazureanu, Adrian-Cosmin Ilie, Emilia Elena Clej, Adrian Ratiu, Ovidiu Calin Ilie

**Affiliations:** 1 Ear-Nose-Throat Department, “Victor Babeș” University of Medicine and Pharmacy Timișoara, 300041 Timișoara, Romania; tischer.alina@umft.ro; 2 Department I—Discipline of Anatomy and Embryology, Faculty of Medicine, “Victor Babeș” University of Medicine and Pharmacy Timișoara, 2nd Eftimie Murgu Square, 300041 Timișoara, Romania; 3 Faculty of Medicine, “Victor Babeș” University of Medicine and Pharmacy, Eftimie Murgu Square 2, 300041 Timișoara, Romania; petra.lazureanu@student.umft.ro; 4 Department of Public Health and Sanitary Management, “Victor Babeș” University of Medicine and Pharmacy, Eftimie Murgu Square 2, 300041 Timișoara, Romania; ilie.adrian@umft.ro; 5 Doctoral School, Department of General Medicine, “Victor Babeș” University of Medicine and Pharmacy, Eftimie Murgu Square 2, 300041 Timișoara, Romania; emilia.clej@umft.ro; 6 Department of Obstetrics and Gynecology, “Victor Babeș” University of Medicine and Pharmacy, Eftimie Murgu Square 2, 300041 Timișoara, Romania; ratiu.adrian@umft.ro; 7 Department I, Discipline of Dental Prostheses Technology (Dental Technology), Faculty of Dental Medicine, “Victor Babeș” University of Medicine and Pharmacy, 300041 Timișoara, Romania; ilie.ovidiu@umft.ro

**Keywords:** type 2 diabetes mellitus, carotid intima–media thickness, visceral adiposity, insulin resistance, hypertension, cardiovascular risk, HOMA-IR

## Abstract

*Background and Objectives*: Type 2 diabetes mellitus (T2DM), visceral adiposity, and hypertension frequently coexist, but their independent associations with carotid intima–media thickness (cIMT) remain uncertain. We compared cardiometabolic profiles by diabetes status and evaluated adjusted associations with cIMT. *Materials and Methods*: This retrospective cross-sectional study included 147 adults admitted to Clinical CF Hospital Timișoara between 1 July 2025 and 30 June 2026. Bilateral cIMT was available in 141 patients. Group comparisons, Spearman correlation, and heteroscedasticity-robust multivariable linear regression were performed. *Results*: T2DM was present in 39 patients (26.5%), hypertension in 125 (85.0%), and obesity in 120 (81.6%). Compared with participants without diabetes, those with T2DM had higher homeostasis model assessment of insulin resistance (HOMA-IR; 4.11 vs. 3.08; *p* = 0.005) and fasting glucose (6.60 vs. 5.06 mmol/L; *p* < 0.001), but lower total cholesterol (205.2 vs. 225.9 mg/dL; *p* = 0.009) and non-high-density lipoprotein cholesterol (156.5 vs. 176.6 mg/dL; *p* = 0.006). These lower lipid values may reflect treatment or other residual confounding and should not be interpreted as an intrinsic T2DM phenotype. Mean cIMT was numerically higher in T2DM (0.619 vs. 0.592 mm; *p* = 0.284). Visceral fat area correlated with cIMT before adjustment (*ρ* = 0.237; *p* = 0.005), but not after adjustment. Age (*β* = 0.057 mm/decade; *p* < 0.001) and hypertension (*β* = 0.046 mm; *p* = 0.042) retained adjusted associations with cIMT. *Conclusions*: T2DM identified metabolic dysregulation, whereas age and hypertension showed the strongest adjusted associations with cIMT. Visceral adiposity showed an unadjusted but not independent association, supporting comprehensive cardiovascular risk assessment and rigorous blood pressure management.

## 1. Introduction

Type 2 diabetes mellitus (T2DM) is a major accelerator of atherosclerotic cardiovascular disease and frequently coexists with obesity, hypertension, dyslipidemia, and chronic kidney disease. Contemporary European and American guidance therefore frames diabetes management as a cardiovascular–kidney–metabolic problem rather than an isolated disorder of glucose homeostasis [1,2,3,4,5,6]. Even when conventional risk factors appear controlled, considerable heterogeneity in vascular burden persists among individuals with T2DM [7,8,9,10].

Visceral adipose tissue is biologically distinct from subcutaneous fat. Through increased lipolysis, ectopic lipid flux, adipokine imbalance, oxidative stress, and low-grade inflammation, visceral adiposity can promote insulin resistance, endothelial dysfunction, arterial stiffness, and atherogenesis [11,12,13,14,15]. Body mass index (BMI) alone cannot fully capture this heterogeneity, and direct or device-derived estimates of visceral fat may improve phenotyping of cardiometabolic risk.

Carotid intima–media thickness (cIMT) is a reproducible ultrasound marker of arterial remodeling and cumulative exposure to vascular risk factors. Although cIMT should not be interpreted as synonymous with focal plaque, higher values are associated with future myocardial infarction and stroke at the population level [16,17,18,19,20]. Previous studies have linked cIMT to insulin resistance, visceral adiposity, and adverse cardiometabolic profiles, but the independence and magnitude of these associations vary by population, vascular segment, measurement protocol, and covariate structure [21,22,23,24].

Hospitalized internal medicine populations are clinically relevant because multimorbidity is common, risk-factor control is often incomplete, and metabolic and cardiovascular abnormalities are evaluated simultaneously. However, these patients are underrepresented in tightly selected prevention cohorts. We therefore examined 147 adults hospitalized in a Romanian internal medicine department. The primary objective was to determine whether T2DM and visceral adiposity were independently associated with mean bilateral cIMT. Secondary objectives were to compare metabolic, lipid, anthropometric, and blood pressure profiles by diabetes status and to characterize the unadjusted relation between visceral adiposity and subclinical vascular injury.

## 2. Materials and Methods

### 2.1. Study Design and Setting

We conducted a single-center retrospective cross-sectional study of adults admitted to the Internal Medicine II Department of Clinical CF Hospital Timișoara, Romania, between 1 July 2025 and 30 June 2026. The manuscript was prepared in accordance with the STROBE reporting principles for observational studies.

### 2.2. Study Population, Eligibility Criteria, and Cohort Construction

**Figure 1:**
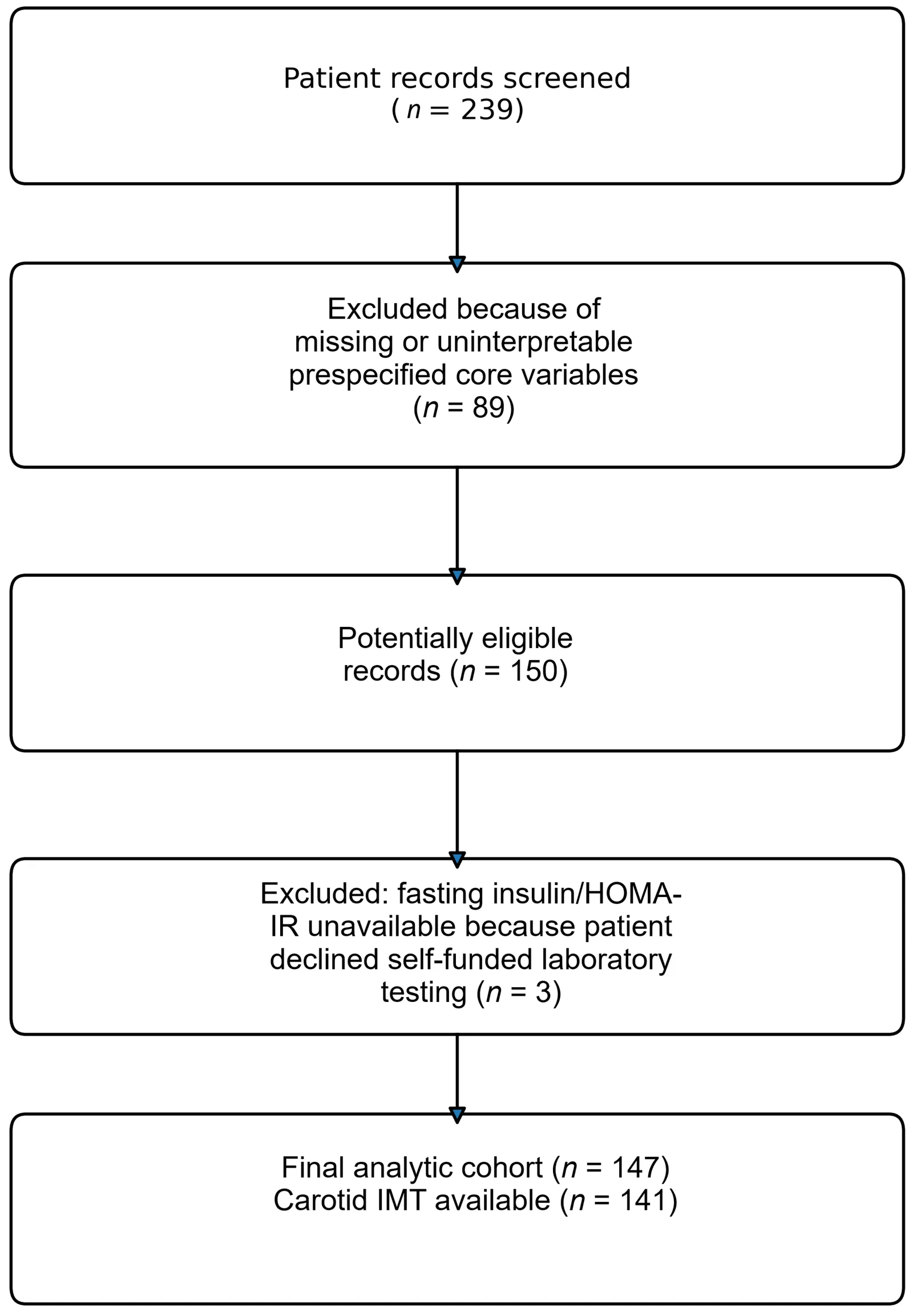
Patient selection and construction of the analytic cohort. Three potentially eligible patients were excluded because fasting insulin testing, required for HOMA-IR calculation, was not performed after they declined the self-funded laboratory investigation.

The source clinical database encompassed admissions to the Internal Medicine II Department between 1 November 2024 and 30 June 2026. For the present analysis, the cohort was restricted a priori to admissions between 1 July 2025 and 30 June 2026, the period during which bioelectrical impedance and carotid ultrasound assessments were consistently available. Within this restricted dataset, 239 patient-level records were screened. Eligible participants were adults with one analyzable record and complete data for age, sex, documented diabetes status, hypertension status, systolic and diastolic blood pressure, fasting glucose, total cholesterol, triglycerides, HDL-C, BMI, and visceral fat area. Eighty-nine of the 239 screened records (37.2%) were excluded because at least one prespecified core variable was missing or uninterpretable, leaving 150 potentially eligible patients. Because the screening log retained the reason for exclusion but did not retain a standardized research extract of demographic and clinical variables for excluded records, a valid included-versus-excluded comparison could not be performed. The missingness mechanism therefore could not be empirically classified as missing completely at random. Complete-case analysis was selected because the primary exposures and principal confounders were required to define a common analytic cohort, and the available record count did not support reliable multiple imputation under unverifiable assumptions. This approach may nevertheless introduce selection bias and reduce generalizability. Fasting insulin, required for HOMA-IR, is not covered by the hospital or by the national health insurance scheme; it was therefore offered as an optional, self-funded determination performed at Bioclinica, an accredited private laboratory in Timisoara, Romania. Three of the 150 patients chose not to undergo this determination and consequently had no calculable HOMA-IR value, leaving a final analytic cohort of 147 patients. This cost-dependent step may have introduced socioeconomic selection. No patient identifiers were used in the statistical dataset. Bilateral cIMT values were available in 141 of the 147 included patients (Figure 1).

### 2.3. Clinical and Laboratory Variables

Age, sex, smoking status, documented type 2 diabetes mellitus (T2DM), hypertension, antihypertensive treatment, lipid-lowering therapy, blood pressure, fasting glucose, fasting insulin, total cholesterol, triglycerides, high-density lipoprotein cholesterol (HDL-C), low-density lipoprotein cholesterol (LDL-C), body weight, height, waist circumference, body mass index (BMI), and visceral fat area were extracted from the source clinical database. Blood pressure was measured using a calibrated automated sphygmomanometer Omron HEM-907XL (Omron Healthcare, Kyoto, Japan), following routine institutional procedures. Non-high-density lipoprotein cholesterol (non-HDL-C) was calculated as total cholesterol minus HDL-C. Obesity was defined as BMI ≥ 30 kg/m^2^. Current smoking was distinguished from former and never smoking using mutually exclusive smoking status fields in the database. T2DM and hypertension were defined from diagnoses documented by the treating physicians in the medical record and/or ongoing disease-specific treatment recorded at admission; no study-specific diagnostic adjudication or repeat confirmatory testing was undertaken.

Fasting blood samples were obtained after an overnight fast of at least 8 h. Fasting plasma glucose, total cholesterol, triglycerides, HDL-C, and LDL-C were determined using an automated clinical chemistry analyzer (Roche Cobas c 501, Roche Diagnostics, Mannheim, Germany) as part of routine inpatient care, whereas fasting insulin was measured using an automated immunoassay platform (Roche Cobas e 411, Roche Diagnostics, Mannheim, Germany). Fasting insulin is not reimbursed by the hospital or by the national health insurance scheme and was therefore determined only in patients who opted for it and covered its cost, at Bioclinica, an accredited private laboratory in Timișoara, Romania, according to the laboratory’s standardized analytical procedures and internal quality-control protocols. The anonymized clinical and laboratory data used in the present retrospective analysis were handled in accordance with the institutional ethics approval described in Section 2.7. Homeostasis model assessment of insulin resistance (HOMA-IR) was subsequently calculated using the conventional formula: fasting insulin (µU/mL) × fasting plasma glucose (mmol/L)/22.5 [25,26].

Diabetes status was based on the documented clinical diagnosis and/or glucose-lowering treatment recorded in the medical record. Hypertension was similarly based on a documented diagnosis and/or antihypertensive treatment recorded at admission. Standardized study-specific diagnostic adjudication was not performed. A standardized glycated hemoglobin (HbA1c) measurement was not available for the entire cohort; consequently, HbA1c was not included in the present analysis.

### 2.4. Anthropometry and Visceral Adiposity

BMI was calculated as weight divided by height squared. In accordance with the World Health Organization classification, BMI 25.0–29.9 kg/m^2^ denotes overweight, and BMI ≥ 30.0 kg/m^2^ denotes obesity; obesity was therefore defined as BMI ≥ 30 kg/m^2^ in this analysis [27]. Waist circumference was recorded during the index hospitalization. Visceral fat area was assessed using a multi-frequency bioelectrical impedance analyzer (InBody 770, InBody Co., Ltd., Seoul, Republic of Korea). Measurements were performed in the morning after an overnight fast of at least 8 h, with patients voiding immediately beforehand, standing barefoot on the device platform in light indoor clothing, and holding the hand electrodes with arms slightly abducted. Metal objects and electronic devices were removed prior to measurement. Visceral fat area (cm^2^) was obtained from the device software according to the manufacturer’s algorithm. Because device-derived visceral fat estimates are method-dependent, visceral fat area was modeled continuously and by cohort-specific tertiles rather than using a universal clinical threshold. Published comparisons support a correlation between multi-frequency BIA estimates and CT-derived visceral adipose tissue, but individual-level agreement is imperfect and can vary across body size and clinical context [28,29,30]. Accordingly, the measurement was treated as a device-derived proxy rather than an anatomic equivalent of CT or MRI.

### 2.5. Carotid Ultrasound

Bilateral carotid IMT values were available in 141 patients. The primary vascular outcome was the arithmetic mean of the recorded left and right carotid IMT measurements. Carotid ultrasound examinations were performed using a high-resolution B-mode ultrasound system (MyLab X8, Esaote, Genoa, Italy) equipped with a linear-array transducer (3–11 MHz or 7–12 MHz). Patients were examined in the supine position with the neck slightly extended, and the head rotated approximately 45° contralaterally. Measurements were obtained from the far wall of the distal common carotid artery, 10 mm proximal to the carotid bulb, in a plaque-free segment, at end-diastole using electrocardiographic (ECG) R-wave gating. Three measurements were taken on each side and averaged. The mean bilateral cIMT was calculated as the arithmetic mean of the left and right values. All examinations were performed by experienced operators according to the Mannheim Carotid Intima–Media Thickness Consensus [18]. cIMT was analyzed as a continuous marker of arterial remodeling; focal plaque was not inferred from cIMT values. The source database did not preserve operator identifiers in a form suitable for estimating the number of operators or calculating intra- or inter-observer reliability. The six absent bilateral cIMT records reflected unavailable or technically non-analyzable routine examinations; the screening dataset did not preserve sufficiently granular reason codes to distinguish these mechanisms or support a reliable formal comparison with the 141 participants with cIMT. These limitations are reported explicitly rather than assuming random outcome missingness.

#### Timing and Inpatient Context

Laboratory sampling, bioimpedance assessment, and carotid ultrasonography were performed during the same index hospitalization as part of routine clinical evaluation. Fasting laboratory sampling and bioimpedance were conducted under the standardized morning conditions described above. The retrospective database did not retain a sufficiently reliable examination timestamp for every modality to confirm that all three assessments occurred on the same calendar day or within a uniform hour-based window. Admission diagnoses and markers of acute-illness severity were not coded consistently enough for stratified analyses. Consequently, acute illness, stress hyperglycemia, altered intake, hydration shifts, and inpatient treatment changes may have influenced contemporaneous glucose, lipid, insulin, and impedance-derived measurements.

### 2.6. Statistical Analysis

Continuous variables are summarized as mean ± standard deviation when approximately symmetric and as median [interquartile range] when skewed. Categorical variables are expressed as counts and percentages. Between-group comparisons were performed using Welch’s *t*-test, the Mann–Whitney U test, Pearson’s chi-square test, or Fisher’s exact test, as appropriate. Spearman correlation quantified unadjusted associations involving visceral fat area.

The primary multivariable association model used mean bilateral cIMT as the continuous dependent variable. Prespecified covariates were age (per 10 years), sex, T2DM, hypertension, current smoking, visceral fat area (per 1 standard deviation), non-HDL cholesterol (per 1 standard deviation), and recorded statin therapy. Visceral fat area was chosen as the primary adiposity exposure because the study question concerned visceral adiposity specifically; BMI and waist circumference were treated as alternative, correlated adiposity proxies and were not entered simultaneously to avoid redundant adjustment and unstable interpretation. HOMA-IR was intentionally excluded from the primary model because it may function as an intermediate variable between adiposity/T2DM and vascular remodeling. Standardization of visceral fat area and non-HDL-C placed their coefficients on a one-SD scale and improved comparability without altering statistical significance. HOMA-IR was right-skewed and therefore log-transformed in the exploratory sensitivity model to reduce leverage from extreme values and better satisfy linear-model assumptions. Pairwise relationships, variance inflation factors, residual-versus-fitted and quantile–quantile plots, and influential observations were reviewed as model diagnostics. These reviews did not identify a major qualitative violation that invalidated linear-model interpretation; HC3 robust standard errors were nevertheless retained to limit sensitivity to heteroscedasticity and leverage. No variable was removed solely on the basis of statistical significance. Exact diagnostic outputs were not retained in the manuscript analysis file and are therefore not reported as reconstructed values. A separate exploratory sensitivity model added log-transformed HOMA-IR to assess coefficient stability, with explicit recognition that this could represent overadjustment rather than estimation of total effects. Covariate selection was based on clinical relevance, availability across the complete-case cohort, and parsimony relative to *n* = 141 with cIMT. Diabetes duration, HbA1c, renal function, albuminuria, previous cardiovascular events, detailed glucose-lowering therapy, lipid-lowering treatment intensity, and antihypertensive drug classes or regimens were not recorded consistently. BMI- or waist-circumference substitution models and the T2DM × visceral fat interaction were not prespecified and were not performed. Without the original patient-level analytic file, additional coefficients cannot be reconstructed validly from aggregate tables; these analyses should be regarded as priorities for a prospectively specified validation study. Ordinary least-squares regression with HC3 heteroscedasticity-robust standard errors was used because HC3 provides more conservative small-sample inference when residual variance is nonconstant or high-leverage observations are present. Two-sided *p* values < 0.05 were considered statistically significant. Analyses were performed using Python 3 with SciPy and statsmodels.

Post hoc precision was summarized using approximate minimum detectable coefficients at 80% power and a two-sided alpha of 0.05, calculated from each coefficient’s observed robust standard error as (1.96 + 0.84) × SE. These values are descriptive, model-dependent precision metrics rather than prospective power calculations and should not be used to reinterpret nonsignificant findings as equivalence.

### 2.7. Ethics Statement

The study was conducted in accordance with the Declaration of Helsinki. The clinical and laboratory data analyzed in the present retrospective study had been generated during the patients’ hospital care and associated clinical evaluations. All patients had provided written informed consent at the time of hospital admission, including authorization for the anonymized use of their clinical data for scientific research. The research protocol entitled “Systemic Inflammation and Endothelial Dysfunction as Determinants of Cardiovascular Risk in Patients with Comorbidities” received approval from the Ethics Council of Clinical CF Hospital Timișoara (approval no. 5958/30 July 2026). The approval document specifies a study period from 1 November 2024 to 30 June 2026 in the Internal Medicine II Department. The present retrospective analysis was restricted to admissions from 1 July 2025 through 30 June 2026. No additional patient intervention or prospective collection of study-specific data was undertaken for the present analysis. The analytical dataset was fully de-identified before statistical processing.

## 3. Results

### 3.1. Cohort Characteristics

The final cohort comprised 147 patients with a mean age of 55.0 ± 10.1 years; 76 (51.7%) were men. T2DM was documented in 39 patients (26.5%), hypertension in 125 (85.0%), obesity in 120 (81.6%), and current smoking in 28 (19.0%). The mean BMI was 34.1 ± 4.9 kg/m^2^, and the median visceral fat area was 173.5 [154.2–197.1] cm^2^. The mean bilateral cIMT was 0.599 ± 0.121 mm among 141 patients with available ultrasound measurements, as shown in Table 1.

### 3.2. Cardiometabolic Profile According to Diabetes Status

**Figure 2:**
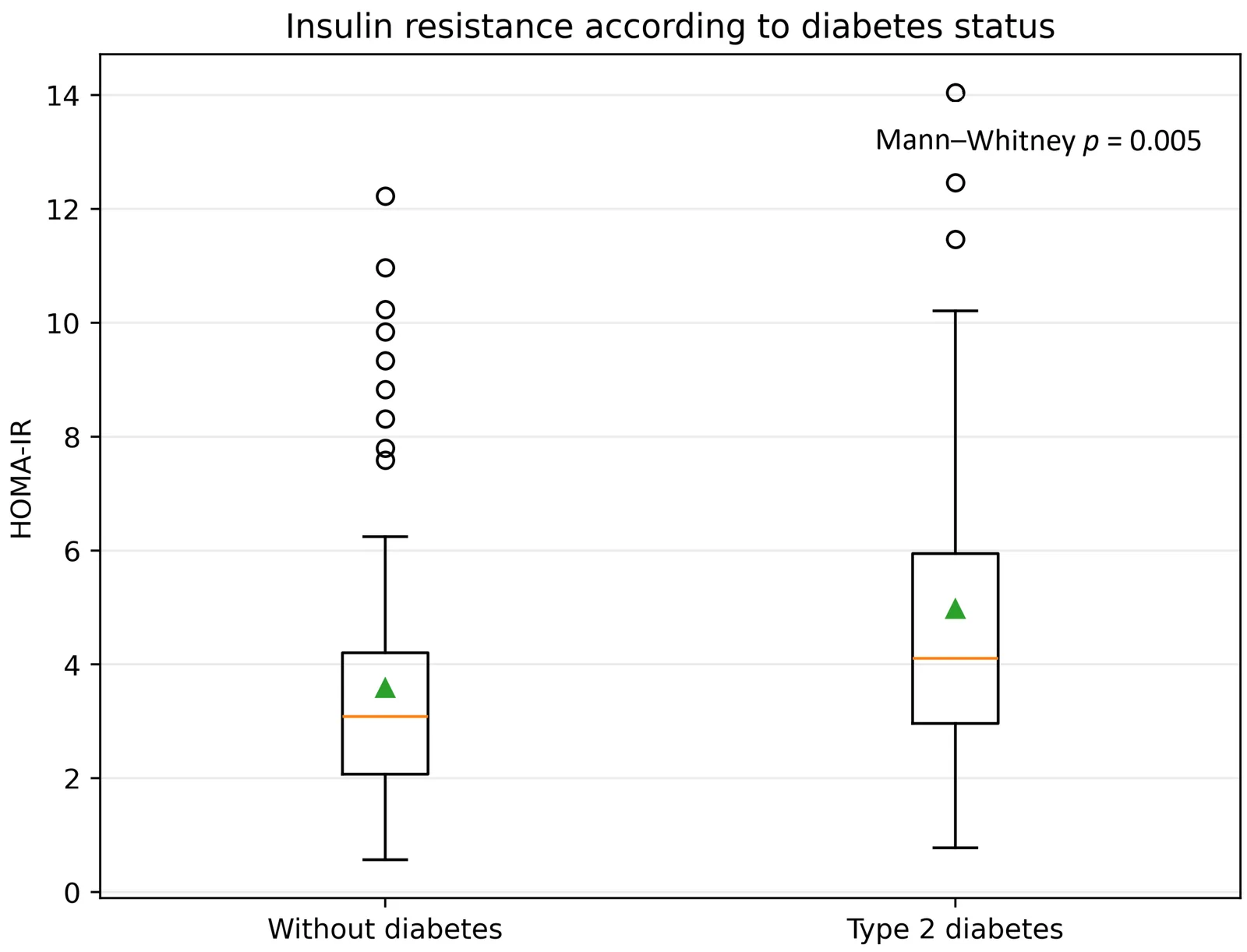
Distribution of HOMA-IR on the original (untransformed) scale according to diabetes status (no diabetes, *n* = 108; T2DM, *n* = 39). Horizontal lines within boxes indicate medians, boxes represent interquartile ranges, whiskers extend to non-outlying observations, circles indicate outliers, and triangles indicate means. The between-group difference was assessed using the Mann–Whitney U test (*p* = 0.005).

Patients with T2DM exhibited a significantly more pronounced insulin-resistant and dysglycemic phenotype than those without diabetes. Median HOMA-IR was significantly higher in patients with T2DM than in those without diabetes (4.11 [2.96–5.95] vs. 3.08 [2.07–4.21], respectively; Mann–Whitney *p* = 0.005), as illustrated in Figure 2. Fasting glucose was also markedly higher in the T2DM group (6.60 [6.28–8.94] vs. 5.06 [4.52–5.47] mmol/L; *p* < 0.001).

In contrast, total cholesterol and non-HDL-C were significantly lower in patients with T2DM, whereas LDL-C, triglycerides, HDL-C, systolic and diastolic blood pressure, BMI, waist circumference, visceral fat area, obesity prevalence, and smoking status did not differ significantly between groups. Recorded statin therapy was numerically more frequent among patients with T2DM (25.6% vs. 15.7%), although this difference did not reach statistical significance. Notably, statin therapy was documented in only 27 of 147 participants (18.4%) despite the cohort’s high cardiovascular-risk profile; incomplete medication documentation, non-treatment, intolerance, or nonadherence could not be distinguished. Mean cIMT was numerically higher in the T2DM group (0.619 ± 0.135 mm vs. 0.592 ± 0.116 mm), but the difference was not statistically significant (*p* = 0.284).

### 3.3. Visceral Adiposity and Carotid IMT

**Figure 3:**
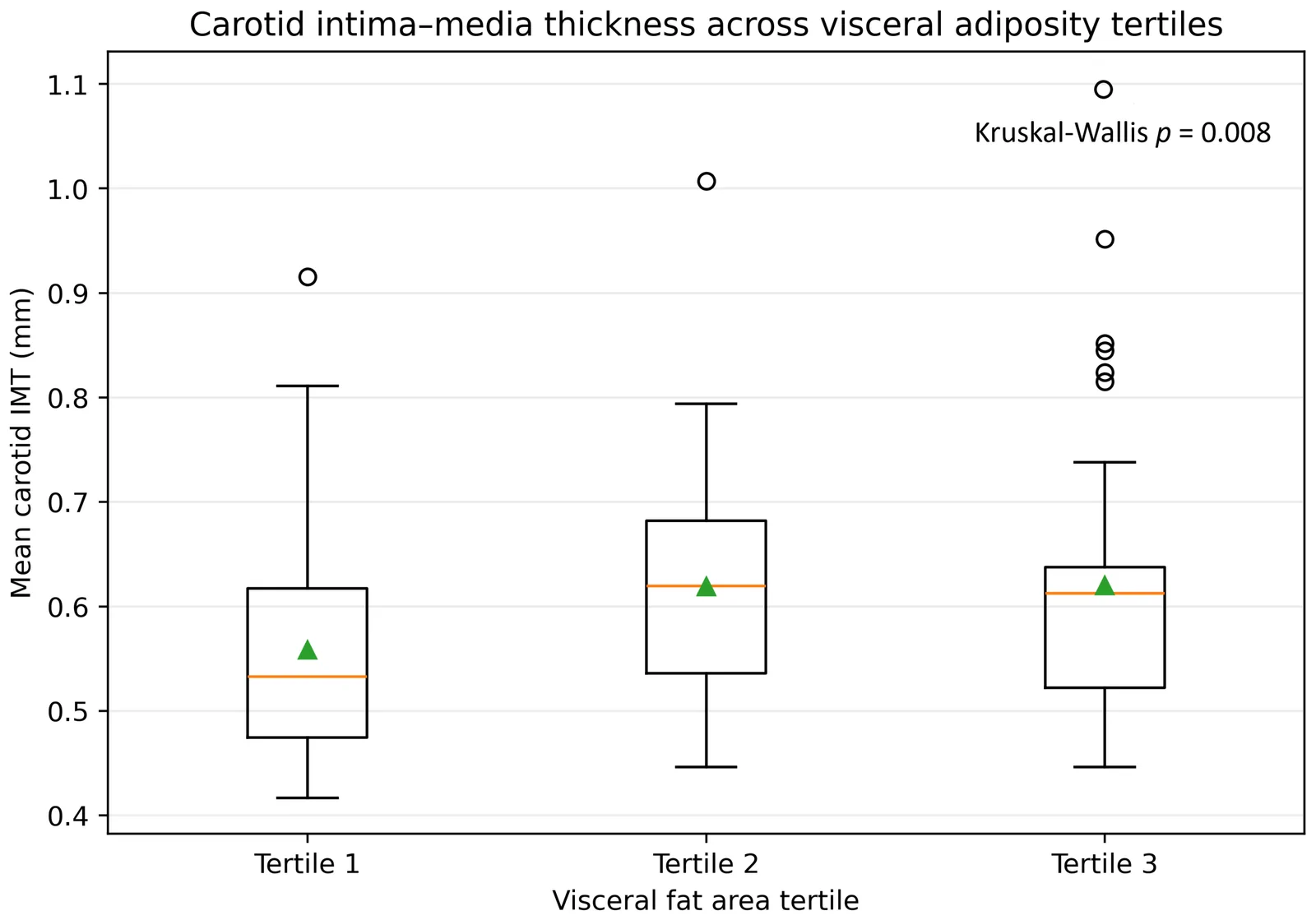
Mean carotid intima–media thickness across cohort-specific visceral-adiposity tertiles. cIMT sample sizes were *n* = 48, *n* = 47, and *n* = 46 in tertiles 1, 2, and 3, respectively. Horizontal lines within boxes indicate medians, boxes represent interquartile ranges, whiskers extend to non-outlying observations, circles indicate outliers, and triangles indicate means. The overall between-tertile difference was assessed using the Kruskal–Wallis test (*p* = 0.008).

Visceral fat area correlated positively with mean cIMT (Spearman *ρ* = 0.237; *p* = 0.005). Mean cIMT increased from 0.558 ± 0.106 mm in the lowest visceral fat tertile to 0.619 ± 0.110 mm and 0.620 ± 0.137 mm in the middle and highest tertiles, respectively (Kruskal–Wallis *p* = 0.008). This pattern suggested a threshold-like unadjusted relationship rather than a strictly linear dose–response association (Table 2, Figure 3).

### 3.4. Multivariable Adjusted Associations with Carotid IMT

**Figure 4:**
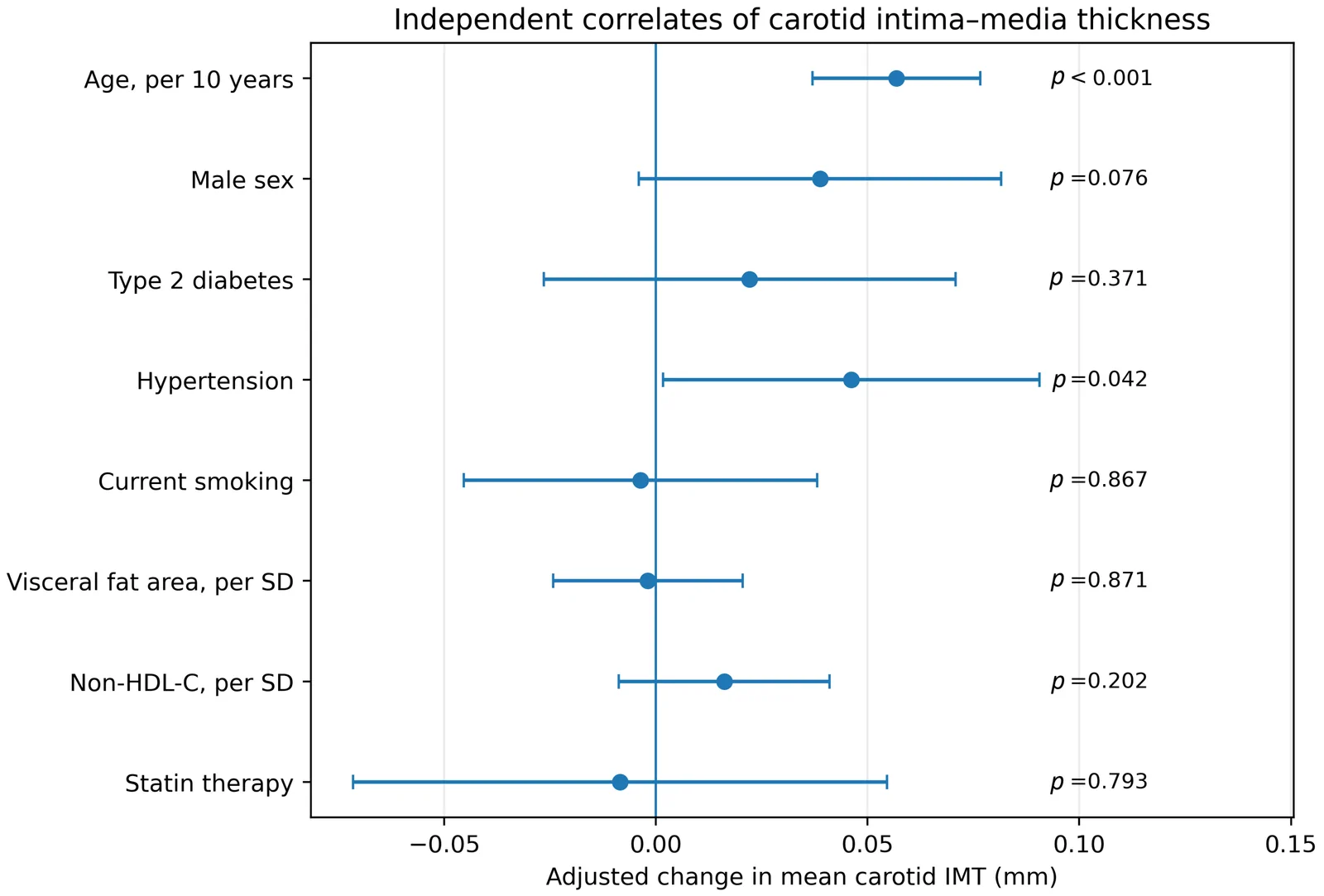
Adjusted coefficients for mean carotid intima–media thickness in the primary multivariable model (*n* = 141). Points represent regression coefficients (beta), while horizontal lines indicate 95% confidence intervals, and the vertical reference line denotes no adjusted difference (beta = 0). Visceral fat area and non-HDL-C are shown per 1-SD increase.

The multivariable association model included 141 patients (R^2^ = 0.292; adjusted R^2^ = 0.249). Each 10-year increase in age was associated with a 0.057-mm higher cIMT (95% confidence interval (CI) 0.037 to 0.077; *p* < 0.001). Hypertension was associated with a 0.046 mm higher cIMT after covariate adjustment (95% CI 0.002 to 0.091; *p* = 0.042). Male sex showed a borderline adjusted association. T2DM, current smoking, visceral fat area, non-HDL cholesterol, and statin therapy did not show statistically significant adjusted associations with cIMT in this sample, as shown in Table 3 and Figure 4. HOMA-IR was not part of the primary model. In a separate exploratory sensitivity model, adding log-transformed HOMA-IR did not materially change the overall coefficient pattern, and HOMA-IR was not statistically significant. Because insulin resistance may lie on the causal pathway linking visceral adiposity or T2DM to arterial remodeling, this sensitivity model was considered an overadjusted robustness analysis rather than evidence against mediated effects. No formal mediation analysis was performed. Based on the observed robust confidence intervals, the approximate minimum detectable coefficient at 80% power was about 0.069 mm for T2DM and 0.032 mm per 1-SD increase in visceral fat area. Thus, smaller independent associations could readily have gone undetected; the nonsignificant estimates are not evidence of equivalence or absence of association.

## 4. Discussion

In this single-center cohort of 147 hospitalized adults, three findings were most clinically relevant. First, T2DM identified a markedly dysglycemic and insulin-resistant phenotype but did not show a statistically significant adjusted association with cIMT after accounting for age, hypertension, adiposity, smoking, and atherogenic cholesterol. Second, visceral fat area was associated with cIMT in unadjusted analyses, and cIMT was higher in the two upper visceral-fat tertiles; however, the association was almost completely attenuated by age and other conventional covariates. Third, age and hypertension emerged as the strongest adjusted associations with carotid arterial remodeling.

The diabetic group displayed an apparent metabolic–lipid discordance: fasting glucose and HOMA-IR were higher, whereas total and non-HDL cholesterol were lower. This should not be interpreted as intrinsically favorable vascular biology in T2DM. Contemporary cohort evidence shows that the excess cardiovascular risk of T2DM is strongly influenced by the cumulative control of glycemia, blood pressure, lipids, smoking, and renal markers [7,8,9,10]. Lower cholesterol in the present diabetic subgroup may reflect treatment selection, dietary changes after diagnosis, survival or referral patterns, unrecorded combination lipid-lowering therapy, or residual confounding. The finding that cIMT remained numerically higher despite lower atherogenic cholesterol is compatible with the concept that vascular burden integrates lifetime exposure rather than a single inpatient lipid measurement. Statin exposure was available only as a binary recorded variable; dose, intensity, treatment duration, adherence, and non-statin lipid-lowering therapy were not captured consistently. Consequently, the model could not distinguish low- from high-intensity treatment or cumulative exposure. This coarse adjustment may have left substantial residual confounding in both the lower cholesterol values observed in T2DM and the statin–cIMT coefficient. Contemporary meta-analytic evidence that statin treatment can influence cIMT further supports cautious interpretation of this binary covariate [31].

Visceral adiposity correlated with cIMT before adjustment, consistent with extensive literature linking abdominal fat distribution to insulin resistance, dyslipidemia, inflammation, and vascular dysfunction [11,12,13,14,15,21,22,23,24]. A large 2025 analysis combining MRI-derived visceral adipose tissue with carotid MRI in CAHHM and ultrasound cIMT in the UK Biobank likewise found positive associations that were attenuated after adjustment for conventional cardiovascular risk factors [32]. In the present study, the adjusted coefficient was null. This attenuation is biologically plausible because insulin resistance may mediate part of the pathway from visceral adiposity and T2DM to vascular remodeling, whereas age, blood pressure, and visceral adiposity also share interrelated causal pathways. Importantly, HOMA-IR was not included in the primary model; it was added only in a separate sensitivity analysis. Conditioning on a potential mediator can constitute overadjustment and reduce the apparent total association of upstream exposures. Therefore, the sensitivity analysis cannot be interpreted as showing that visceral adiposity or T2DM is biologically unimportant, and the cross-sectional design does not permit formal temporal or causal mediation inference. The results support distinguishing total, direct, and mediated pathways in future longitudinal studies rather than treating statistical attenuation as an absence of biological effect.

The InBody 770 provides an algorithm-derived estimate of visceral fat area rather than direct anatomic quantification. CT and MRI remain the reference imaging methods because they directly delineate visceral and subcutaneous compartments. Validation studies indicate that InBody 770-derived visceral fat estimates correlate with CT-derived visceral adipose tissue, but concordance is only moderate overall and may vary by sex, body size, and population [28]. Other comparisons of BIA with CT have reported limited individual-level agreement and possible systematic under- or overestimation, particularly at higher adiposity levels [29]. Moreover, impedance-based estimates depend on proprietary prediction equations and assumptions regarding body geometry and hydration; food intake, recent exercise, fluid and electrolyte disturbances, skin temperature, and posture can affect measurements. Standardized fasting, pre-measurement voiding, clothing, electrode contact, and positioning in the present study were intended to reduce short-term variability, but they cannot eliminate method-related error. Recent real-world evaluation of the InBody 770 found high repeatability for several body-composition measures but weaker validity for visceral adipose tissue estimates, highlighting that reliability does not necessarily imply criterion accuracy [30]. Accordingly, our visceral fat area values are best interpreted as standardized, device-specific estimates suitable for within-cohort ranking and association analyses, not as interchangeable with CT- or MRI-derived absolute visceral fat area.

The independent association between hypertension and cIMT reinforces the importance of cumulative pressure load. Hypertension was present in 85% of the cohort, creating a high-risk background in both diabetic and non-diabetic patients. Current ESC recommendations emphasize earlier recognition of elevated blood pressure, standardized measurement, and intensive multifactorial risk reduction, particularly in patients with diabetes or target-organ damage [2,3,4,5]. In this setting, the 0.046 mm adjusted difference associated with hypertension is directionally consistent with cumulative pressure-related arterial remodeling; however, cIMT is not itself a treatment target, and the cross-sectional estimate should not be interpreted as a causal effect size.

The absence of a statistically significant adjusted T2DM–cIMT coefficient in this sample does not negate the cardiovascular importance of diabetes or the long-standing epidemiological evidence linking T2DM to excess coronary risk [33]. Rather, it may reflect limited precision, treatment-related confounding, mediation through insulin resistance and other cardiometabolic pathways, or the pervasive obesity and hypertension of this inpatient cohort. The confidence interval around the diabetes coefficient remained compatible with both a modest decrease and a clinically relevant increase, and the study was not powered to exclude subtle associations. Diabetes duration, HbA1c, albuminuria, kidney function, microvascular complications, glucose-lowering therapy, and prior cardiovascular events were unavailable for complete adjustment. Recent meta-analytic evidence continues to show greater cIMT in T2DM populations [24], while a 2024 network meta-analysis indicates that specific glucose-lowering therapies may differentially influence cIMT progression [34], reinforcing the potential for unmeasured treatment confounding in our analysis.

These findings fit the emerging cardiovascular–kidney–metabolic framework, which emphasizes the interconnected progression from excess adiposity and metabolic dysfunction to subclinical and clinical cardiovascular disease [6,7]. For clinical practice, the data support a layered assessment: confirm glycemic status and insulin resistance; quantify obesity and central adiposity; aggressively evaluate blood pressure; interpret lipid values in the context of therapy and prior exposure; and use vascular imaging only when it addresses a defined clinical or research question. A single apparently favorable parameter, such as lower cholesterol during hospitalization, should not be assumed to neutralize accumulated vascular risk. More broadly, the multifactorial interpretation of these findings is consistent with global evidence on modifiable myocardial infarction risk [35], contemporary European risk-estimation frameworks [36], and ESC/EAS lipid-management guidance emphasizing total cardiovascular risk rather than isolated lipid measurements [5,37]. The clustering of central adiposity, dysglycemia, hypertension, and dyslipidemia is also consistent with the metabolic-syndrome construct [38].

Several aspects strengthen the analysis: transparent cohort construction; simultaneous use of metabolic, anthropometric, lipid, blood pressure, medication, and ultrasound variables; use of bilateral mean cIMT; standardized continuous modeling of visceral adiposity; and HC3 robust standard errors. The study also reflects a real-world internal medicine population with a high prevalence of multimorbidity. This increases relevance to similar hospitalized high-risk patients but does not confer broad population representativeness.

### Limitations

This study should be interpreted in light of several limitations. First, the retrospective cross-sectional design precludes conclusions regarding temporality or causality; all reported relationships are associations rather than direct effects. Second, this was a single-center inpatient cohort with high prevalences of obesity and hypertension, limiting statistical precision and external validity for community populations, younger adults, and lower-risk settings. Restricted cardiometabolic variability may also have reduced the ability to separate correlated independent associations. Complete-case selection was substantial: In total, 89 of 239 screened records (37.2%) were excluded because prespecified core variables were incomplete or uninterpretable. A standardized research extract for excluded records was not retained, so included-versus-excluded comparisons and empirical classification of the missingness mechanism were not possible. Complete-case analysis avoided imputation based on unverifiable assumptions but may have introduced selection bias. Three additional potentially eligible patients lacked HOMA-IR after declining an optional, self-funded insulin test; willingness or ability to pay may therefore have introduced socioeconomic selection. Third, residual confounding is substantial. Diabetes duration, HbA1c, renal function, albuminuria, inflammatory biomarkers, cardiovascular history, microvascular complications, and complete treatment exposure were unavailable. Statin therapy was captured only as a binary field; dose, intensity, duration, adherence, and non-statin therapy were unavailable. Antidiabetic and antihypertensive drug class, dose, combination regimen, duration, and adherence—including GLP-1 receptor agonists and SGLT2 inhibitors—were not consistently recorded. T2DM and hypertension relied on routine clinical documentation rather than study-specific adjudication, allowing exposure misclassification. Fourth, visceral fat area was estimated by BIA rather than by CT or MRI. Despite standardized fasting, voiding, clothing, and positioning, the proprietary estimate remains sensitive to hydration, body geometry, acute illness, and population-specific prediction error. Nondifferential error could attenuate a true association, while systematic error could bias it unpredictably. Fifth, although carotid acquisition followed a standardized protocol, operator identifiers and formal intra- and inter-observer reproducibility were unavailable. Six participants lacked analyzable bilateral cIMT; granular reason codes and a formal comparison with those having cIMT were unavailable, so outcome missingness cannot be assumed random. Focal plaque was not systematically quantified. Sixth, the modest sample size, the 39-person T2DM subgroup, and wide confidence intervals limited power. The approximate 80-power minimum detectable coefficients were 0.069 mm for T2DM and 0.032 mm per SD of visceral fat area; smaller clinically relevant associations cannot be excluded. Nonsignificance therefore represents failure to demonstrate an independent association, not evidence of no association. Interaction and alternative adiposity models would have limited precision and were not prespecified. Seventh, laboratory, BIA, and ultrasound measurements occurred during the index hospitalization but could not be aligned to a uniform same-day window retrospectively. Acute illness, stress responses, altered intake, hydration shifts, and treatment changes may have affected glucose, insulin, lipids, and BIA estimates. Admission diagnoses and acute-illness severity were not consistently coded for stratification. Finally, secondary comparisons were exploratory and were not adjusted for multiplicity. Larger prospectively assembled, multicenter cohorts should preserve screening variables, standardize timing, use CT/MRI adiposity measures, quantify ultrasound reproducibility, comprehensively capture renal and treatment covariates, and prespecify alternative-adiposity, interaction, mediation, and longitudinal analyses.

## 5. Conclusions

Among 147 adults hospitalized in an internal medicine department, T2DM was associated with pronounced dysglycemia and insulin resistance but did not show a statistically significant adjusted association with carotid IMT in this exploratory cross-sectional analysis. Visceral adiposity showed an unadjusted association with carotid arterial remodeling that attenuated after covariate adjustment; this attenuation may reflect shared pathways, mediation through insulin resistance, measurement error, residual confounding, or limited precision and should not be interpreted as an absence of biological importance. Age and hypertension showed the strongest adjusted associations with cIMT. Clinically, these findings support comprehensive cardiovascular risk assessment in high-risk hospitalized adults, with particular attention to accurate blood pressure evaluation and sustained hypertension control rather than reliance on any single metabolic or lipid measurement. These findings are associative and hypothesis-generating. Larger, prospectively assembled cohorts with comprehensive treatment data, imaging-based adiposity assessment, prespecified interaction and mediation analyses, and longitudinal outcomes are required before definitive conclusions about direct or independent effects can be drawn.

## Figures and Tables

**Table 1:** Baseline characteristics according to diabetes status. Values are mean ± SD, median [IQR], or *n* (%). BMI, body mass index; HDL-C, high-density lipoprotein cholesterol; HOMA-IR, homeostasis model assessment of insulin resistance; IMT, intima–media thickness; LDL-C, low-density lipoprotein cholesterol; T2DM, type 2 diabetes mellitus.

Variable	Overall (*n* = 147)	No Diabetes (*n* = 108)	T2DM (*n* = 39)	*p* Value
Age, years	55.0 ± 10.1	54.3 ± 10.4	57.2 ± 9.0	0.102
Male sex	76 (51.7%)	59 (54.6%)	17 (43.6%)	0.237
Hypertension	125 (85.0%)	90 (83.3%)	35 (89.7%)	0.336
Current smoking	28 (19.0%)	23 (21.3%)	5 (12.8%)	0.248
Obesity (BMI ≥ 30 kg/m^2^)	120 (81.6%)	90 (83.3%)	30 (76.9%)	0.376
HOMA-IR	3.24 [2.20–4.78]	3.08 [2.07–4.21]	4.11 [2.96–5.95]	0.005
Fasting glucose, mmol/L	5.31 [4.74–6.30]	5.06 [4.52–5.47]	6.60 [6.28–8.94]	<0.001
Systolic blood pressure, mmHg	141.7 ± 20.9	141.0 ± 21.1	143.7 ± 20.7	0.490
Diastolic blood pressure, mmHg	86.7 ± 11.4	86.8 ± 11.9	86.5 ± 9.8	0.891
Total cholesterol, mg/dL	220.4 ± 48.5	225.9 ± 50.9	205.2 ± 37.6	0.009
Triglycerides, mg/dL	180.0 [133.9–235.8]	182.1 [139.9–233.8]	171.0 [114.5–235.3]	0.233
HDL-C, mg/dL	49.1 ± 12.7	49.3 ± 12.4	48.7 ± 13.8	0.823
LDL-C, mg/dL	131.6 ± 42.0	134.4 ± 43.1	124.5 ± 38.8	0.193
Non-HDL-C, mg/dL	171.2 ± 47.9	176.6 ± 51.4	156.5 ± 32.6	0.006
Statin therapy recorded	27 (18.4%)	17 (15.7%)	10 (25.6%)	0.171
BMI, kg/m^2^	34.1 ± 4.9	33.7 ± 4.3	35.1 ± 6.1	0.188
Waist circumference, cm	110.4 ± 11.3	109.5 ± 10.0	112.8 ± 14.2	0.190
Visceral fat area, cm^2^	173.5 [154.2–197.1]	172.9 [154.3–195.1]	176.8 [153.8–208.0]	0.669
Mean carotid IMT, mm	0.599 ± 0.121	0.592 ± 0.116	0.619 ± 0.135	0.284

**Table 2:** Metabolic and vascular characteristics across cohort-specific visceral fat tertiles. cIMT was available in 48, 47, and 46 patients in tertiles 1, 2, and 3, respectively.

Visceral-Fat Group	*n*	Observed Range, cm^2^	T2DM, *n* (%)	BMI, kg/m^2^	Mean cIMT, mm
Tertile 1	49	107.5–157.6	14 (28.6%)	31.6 ± 4.3	0.558 ± 0.106
Tertile 2	49	158.0–185.5	10 (20.4%)	33.6 ± 4.1	0.619 ± 0.110
Tertile 3	49	188.6–354.4	15 (30.6%)	37.0 ± 4.7	0.620 ± 0.137

**Table 3:** Heteroscedasticity-robust multivariable linear regression for mean bilateral carotid IMT. Visceral fat area and non-HDL-C were standardized; coefficients represent the change per 1 SD. Model (*n* = 141).

Predictor	*β* Coefficient, mm	95% CI	*p* Value
Intercept	0.223	0.121 to 0.326	<0.001
Age, per 10 years	0.057	0.037 to 0.077	<0.001
Male sex	0.039	−0.004 to 0.082	0.076
Type 2 diabetes	0.022	−0.026 to 0.071	0.371
Hypertension	0.046	0.002 to 0.091	0.042
Current smoking	−0.004	−0.045 to 0.038	0.867
Visceral fat area, per SD	−0.002	−0.024 to 0.021	0.871
Non-HDL-C, per SD	0.016	−0.009 to 0.041	0.202
Statin therapy	−0.008	−0.071 to 0.055	0.793

## Data Availability

De-identified clinical and laboratory data supporting the findings of this study are available from the corresponding author upon reasonable request, subject to institutional data-sharing policies.

## Abbreviations

The following abbreviations are used in this manuscript:

BMI	Body mass index
CI	Confidence interval
cIMT	Carotid intima–media thickness
ECG	Electrocardiography
HbA1c	Glycated hemoglobin A1c
HDL-C	High-density lipoprotein cholesterol
HOMA-IR	Homeostasis model assessment of insulin resistance
IMT	Intima–media thickness
IQR	Interquartile range
LDL-C	Low-density lipoprotein cholesterol
non-HDL-C	Non-high-density lipoprotein cholesterol
SD	Standard deviation
T2DM	Type 2 diabetes mellitus
